# INCREASED INCIDENCE OF INJURIES IN THE SÃO PAULO SOCCER CHAMPIONSHIP POST-PANDEMIC

**DOI:** 10.1590/1413-785220243206e282994

**Published:** 2025-01-10

**Authors:** Alexandre Moreira Sales, Paulo Henrique Schmidt Lara, Nathalia Bofill Burger, Raphael Ribeiro Moreira, Moisés Cohen, Gustavo Gonçalves Arliani

**Affiliations:** 1. Universidade Federal de Sao Paulo, Centro de Traumatologia do Esporte CETE, Departamento de Ortopedia e Traumatologia DOT-UNIFESP/EPM, Sao Paulo, SP, Brazil.; 2. Instituto Prevent Senior, Sao Paulo, SP, Brazil.

**Keywords:** Soccer, Pandemics, Trauma in athletes, COVID-19, Sports Medicine, Futebol, Pandemia, Trauma em atletas, COVID-19, Medicina Esportiva

## Abstract

Objective: Soccer shows a high incidence of injuries and its cause is multifactorial. The impacts of the coronavirus pandemic are unknown. This study aims to evaluate the incidence of injuries in the 2023 Campeonato Paulista de Futebol (São Paulo Soccer Championship) and compare it to the championships prior to the pandemic. Methods: This study was conducted by collecting data on injuries among players in the A1 Series of the 2023 São Paulo Soccer Championship. Injuries were recorded by each team’s medical staff via an online questionnaire. The variables included: Type of Field, Weather, Temperature, Distance, Home Advantage, Age, Type and Location of Injury. The primary outcome is to evaluate the incidence of injuries, and the secondary outcomes are to analyze the relationship between the described variables, the observed incidence, and the comparison of data obtained from 2016 to 2019. Results: In 2023, 76 injuries were recorded, an incidence of 22.1 injuries/1,000h. Muscle injuries (46.1%) and sprains (18.4%) were the most common. From 2016 to 2019, there was a decrease in the incidence of injuries per 1,000h, respectively: 24.2; 17.6; 14 and 10.5. Conclusion: the incidence in 2023 was 22.1 injuries/1,000h, which points to an increase compared to the pre-pandemic period. *Level of Evidence II, Comparative Prospective Study*

## RESUMO

Objetivo: O futebol apresenta elevada incidência de lesões e sua causa é multifatorial. Os efeitos da pandemia do coronavírus são desconhecidos. O objetivo deste estudo é avaliar a incidência de lesões no Campeonato Paulista de Futebol 2023 e compará-la aos campeonatos anteriores à pandemia. Métodos: estudo conduzido pela coleta de dados referente a lesões dos jogadores da série A1 do Campeonato Paulista de Futebol 2023. As lesões foram registradas pelos médicos de cada time, através do preenchimento de questionário online. As variáveis foram: Tipo de Gramado, Clima, Temperatura, Distância, Mando de jogo, Idade, Tipo e Local de Lesão. O desfecho primário é avaliar a incidência de lesões e os secundários a relação entre as variáveis descritas, a incidência observada e a comparação dos dados obtidos nos anos de 2016 a 2019. Resultados: Em 2023 ocorreram 76 lesões, uma incidência de 22,1 lesões/1000h. As mais comuns: musculares (46,1%) e entorses (18,4%). Nos anos de 2016 a 2019 houve queda na incidência a cada 1000h de jogo, respectivamente: 24,2; 17,6; 14 e 10,5. Conclusão: a incidência em 2023 foi de 22,1 lesões/1000h. Um aumento, comparado ao período pré-pandemia. *Nível de evidência II, Estudo Prospectivo Comparativo*

## INTRODUCTION

 Soccer is played by approximately 240 million amateur athletes and more than 200,000 professional athletes worldwide. ^1^ ^,^ ^2^ Its popularity is global, and the modality covers different age groups, ethnicities, and genders. At the same time, soccer shows a high incidence of injuries, with an estimated rate of up to 70 injuries per 1,000 hours of play ^2^ . The dynamic nature of the sport also contributes to the injury rate, with rapid movements, accelerations, decelerations, changes of direction, jumps, and considerable physical contact. ^1^ ^,^ ^3^

 Over the years, several studies have analyzed the most prevalent injuries in soccer. International organizations such as the United States and the Union of European Football Associations (UEFA) conduct research on the subject, aiming to develop programs aimed at preventing injuries and reducing soccer-related morbidity ^4^ . Muscle injuries, bruises, and sprains are known to account for 75% of injuries affecting professional players, with most in the lower extremities (60–85%). ^3^ ^,^ ^4^

 A study conducted by the UEFA from 2021–2022 pointed to an incidence of 30.5 (+/−11) per 1,000 hours of play, ^5^ whereas a 2005 Swedish study showed 16 to 28 injuries per 1,000 hours of play. ^6^ Moreover, a higher incidence has been found in shorter competitions, such as in the 2011 Copa America, in which an incidence of 70.7 injuries per 1,000 hours of play was observed. ^2^

 The incidence of sports-related injuries is known to be multifactorial but little is known about the possible impacts of the COVID-19 pandemic ^7^ . In this context, this study aims to compare the last four pre-pandemic soccer seasons (2016–2019) and the first post-pandemic season (2023). 

 The study focuses on the A1 series of the *Campeonato Paulista de Futebol* (São Paulo Soccer Championship) and assesses the incidence of injuries that occurred during the 2023 season, comparing them to the pre-pandemic period. Other influencing factors are also assessed, such as the type of field, temperature, weather, distance, and the age of the players. 

## MATERIALS AND METHODS

A prospective comparative study (Level II evidence) was conducted to investigate injuries in the A1 series of the 2023 São Paulo Soccer Championship. An electronic questionnaire was administered, with a model similar to that used in the 2016–2019 championships after each round played.

Data collection occurred collaboratively, with the physicians of each team being responsible for filling out the questionnaire after each match, reporting the occurrence or absence of injuries resulting from the games. The questionnaires were completed ensuring the anonymization of the data provided.

 All athletes regularly registered in the A1 Series of the 2023 São Paulo Soccer Championship were included in this study. Athletes registered by clubs that did not play at least one match were excluded from the study. The definition of injury adopted followed the study by Fuller et al. ^8^ on the 2005 FIFA/F-MARC Consensus Group, in which an injury is defined as “any physical complaint sustained by a player that results from a football match or football training, irrespective of the need for medical attention or time loss from football activities.” 

 The electronic questionnaire consists of a set of 24 questions, including the same ones used in the 2016 to 2019 seasons, with additions covering the categorical and numerical variables assessed. Encompassing characteristics of the matches and players, such as: age, weather, temperature and distance, as well as information about the type of injury (contusion, sprain, muscle, concussion, blunt injury, dislocation, and fracture). The construction of the questionnaire was based on previous studies, which share the same purpose. ^9^ ^,^ ^10^

 The incidence of injury was estimated based on a metric that enables assessing the risk of injury occurrence according to the number of injuries per 1,000 hours of exposure. ^8^ ^,^ ^11^ The formula below was used to estimate exposure: 

Exposure = number of matches x number of players who started the match (22) × duration of the match in minutes (90) / 60

To estimate the incidence during matches, the formula used was as follows:

Incidence in Matches = Number of Injuries/Exposure × 1000

This study was approved by the Human Research Ethics Committee under number 1,660,701

## STATISTICAL ANALYSIS

Exploratory data analysis was performed by summary measures (mean, standard deviation, minimum, median, maximum, frequency, and percentage) and construction of graphs. The Z test for proportions was employed to compare prevalences. The groups with and without lesions were compared using the Chi-square test (categorical variables) or the Mann-Whitney test (numerical variables).

The level of significance was set at 5% (p value < 0.05).

## RESULTS

**Figure 1.**
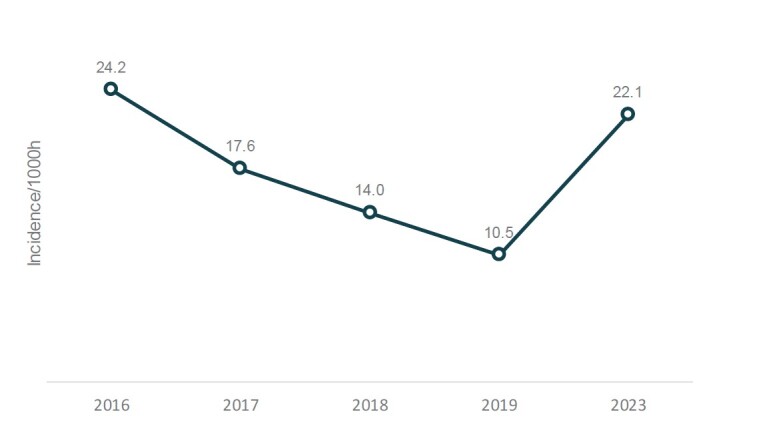
Incidence of injury from 2016 to 2023.

 In the A1 series of the 2023 São Paulo Soccer Championship, 76 injuries were observed in 104 matches ( Table 1 ). The main injuries observed included muscle injuries (strains), representing 46.1%, followed by sprains (18.4%) and concussions (10.5%) ( Table 3 ). In the 104 games of 2019, 36 injuries were observed, which represents a prevalence of 35%, significantly lower (p-value < 0.001) than that found in 2023, of 75%. The incidence in 2019 was 10.5, and in 2023, it was 22.1 injuries per 1,000 hours of play ( Figure 1 ). 

**Table 1.** Comparison between groups (categorical variables – Chi-square test).

Parameter	Total (N=213)	With Injury (n=76)	Withour Injury (n=1,237)	p-value
Type of field:				0.713
Natural	181 (85.0%)	66 (86.8%)	115 (83.9%)	
Synthetic	32 (15.0%)	10 (13.2%)	22 (16.1%)	
Home advantage:				0.617
Home team	103 (48.4%)	39 (51.3%)	64 (46.7%)	
Visiting team	110 (51.6%)	37 (48.7%)	73 (53.3%)	
Weather:				0.093
Sunny	70 (32.9%)	32 (42.1%)	38 (27.7%)	
Cloudy	35 (16.4%)	9 (11.8%)	26 (19.0%)	
Sunshower	7 (3.29%)	2 (2.63%)	5 (3.65%)	
Thunderstorm	2 (0.94%)	2 (2.63%)	0 (0.00%)	
Clean night	62 (29.1%)	17 (22.4%)	45 (32.8%)	
Night with rain	26 (12.2%)	11 (14.5%)	15 (10.9%)	
Rainy	11 (5.16%)	3 (3.95%)	8 (5.84%)	
Match location				0.844
House	103 (48.4%)	39 (51.3%)	64 (46.7%)	
Up to 200 km	70 (32.9%)	23 (30.3%)	47 (34.3%)	
200 – 400 km	28 (13.1%)	9 (11.8%)	19 (13.9%)	
Over 400 km	12 (5.63%)	5 (6.58%)	7 (5.11%)	

**Table 2.** Comparison between groups in relation to temperature on the day of the game (Mann-Whitney test).

Statistic	Total (N=213)	With Injury (n=76)	Without Injury (n=137)	p-value
Mean	25.5	26.1	25.2	0.056
Standard deviation	3.2	2.7	3.4	
Minimum	17	19	17	
Median	26	26	25	
Maximum	32	32	32	

**Table 3.** Characteristics of matches and players with injuries in the 2023 São Paulo Soccer Championship.

Parameter	N (%)		Parameter	N (%)
Age >27 years			Punishment:	
No	35 (46.1%)		Nothing	67 (88.2%)
Yes	41 (53.9%)		Foul, no card	4 (5.26%)
Position:			Foul, yellow card	4 (5.26%)
Defender	16 (21.1%)		Foul, red card	1 (1.32%)
Side midfielder	15 (19.7%)		Injury site:	
Center back	15 (19.7%)		Thigh	32 (42.1%)
Forward	15 (19.7%)		Head/Face	13 (17.1%)
Midfielder	10 (13.2%)		Ankle	11 (14.5%)
Goalkeeper	5 (6.58%)		Knee	8 (10.5%)
Time of play:			Leg	4 (5.26%)
0–15 minutes	8 (10.5%)		Foot	3 (3.95%)
16–30 minutes	13 (17.1%)		Shoulder	3 (3.95%)
31–45 minutes	14 (18.4%)		Hip	1 (1.32%)
46–60 minutes	15 (19.7%)		Abdomen	1 (1.32%)
61–75 minutes	14 (18.4%)		Side:	
76–90 minutes	8 (10.5%)		Right	33 (43.4%)
Extra time 1st period	1 (1.32%)		Left	27 (35.5%)
Extra time 2nd period	3 (3.95%)		Both	2 (2.63%)
Contact:			Not applicable	14 (18.4%)
Yes	37 (48.7%)		Type of injury:	
No	39 (51.3%)		Stretch	35 (46.1%)
“Contra a(o):”			Sprain	14 (18.4%)
1	1 (1.32%)		Concussion	8 (10.5%)
2	5 (6.58%)		Contusion	7 (9.21%)
3	31 (40.8%)		Blunt injury	4 (5.26%)
4	33 (43.4%)		Overuse Injuries	4 (5.26%)
5	6 (7.89%)		Fracture	3 (3.95%)
Foul:			Dislocation	1 (1.32%)
Yes	9 (11.8%)			
No	67 (88.2%)			

 There is also a decrease in the incidence per 1,000 hours of play from 2016 to 2019 and an increase in 2023. From 2016 to 2019, the mean incidence per 1,000 hours of play was 16.5 ( Figure 1 ). 

Table 1 shows the comparison between the matches with and without injuries. No statistically significant differences were found between the groups for the type of field (p-value = 0.713), home advantage (p-value = 0.617), weather (p-value = 0.093), and match location (p-value = 0.844). Table 2 shows that no significant differences were found between the temperature of the games with and without injury (p-value = 0.056). 

Table 3 presents the profile of players and games with at least one injury in the 2023 São Paulo Soccer Championship. 

Among the 76 players who suffered an injury, 41 (53.9%) are over 27 years old. This proportion is not statistically different from 50% (p-value = 0.566).

## DISCUSSION

 In the 2023 season, 76 injuries were recorded, whereas 36 injuries were found in 2019 across the 104 championship matches, resulting in prevalences of 75% and 35%, respectively. The incidence of injuries also showed change, with 10.5 injuries per 1,000 hours of play in 2019 and a significant increase to 22.1 injuries per 1,000 hours of play in 2023. The data found in the 2023 season are consistent with the current literature, which mostly presents a variation of 15 to 30 injuries per 1,000 hours of practice. ^4^ ^,^ ^10^ ^,^ ^5^ However, the increase in incidence suggests an analysis of the possible influences, in particular the role of the pandemic. 

 In the years 2016 to 2019, it is possible to observe a downward trend in the incidence of injuries, ^10^ contrasting with the increase in 2023. The mean incidence value from 2016 to 2019 (16.5 injuries per 1,000 hours of play) suggests relative stability and control of injuries during this period. However, the reversal of this pattern in 2023 draws attention. 

 Another point evaluated in this study was the type of field (natural vs. synthetic), questioned as a potential risk factor for the occurrence of injuries. The data found in this study are compatible with those found in a recently published Systematic Review and meta-analysis, ^12^ not suggesting impact of the type of field with the incidence of injuries. Other factors such as home advantage, weather, age of the athletes, and match location also showed no association with the increase in injuries. This finding suggests that, although these elements are potentially relevant to the occurrence of injuries, ^9^ they did not show a direct or immediately identifiable correlation with the increase found in 2023. 

The COVID-19 pandemic has introduced a number of changes to the dynamics of the sport, including health protocols, audience restrictions, and changes to schedules and calendars. After its end, most of these adaptations ceased to exist, causing an abrupt increase in the volume of training and games with the resumption of normality in the competition calendar. Factors such as mental health, a tendency toward increased sedentary behavior during the pandemic (isolation), and a sudden increase in external workload are identified as potential contributors to the observed change in patterns.

 The proportion of players who suffered injuries and were over 27 years of age (53.9%) did not present a significant difference when compared to the proportion of players under 27 years of age, different from what has been reported in other studies. ^13^

 Some limitations of this study must be considered. The administered questionnaire does not enable the evaluation of factors such as injury history, medical comorbidities, sleep quality, nutrition, sports experience, stress management, and physical conditioning, which are known to be associated with injury recovery and incidence. ^14^ ^,^ ^15^ Additionally, reliance on the voluntary and accurate participation of club physicians in completing the questionnaire may lead to underreporting of cases. We also know that concussion is an underdiagnosed condition, and may be underreported. ^16^

The fact that it is a competition at the beginning of the season impacts the athletes’ conditioning and, indirectly, the incidence of injuries. In terms of analyzing the type of field, the number of clubs with artificial turf in their stadiums was found to be small, reported for only two out of the 16 participants. This results in few games played under these conditions, hindering the impact assessment of the type of field.

**Figure 2.**
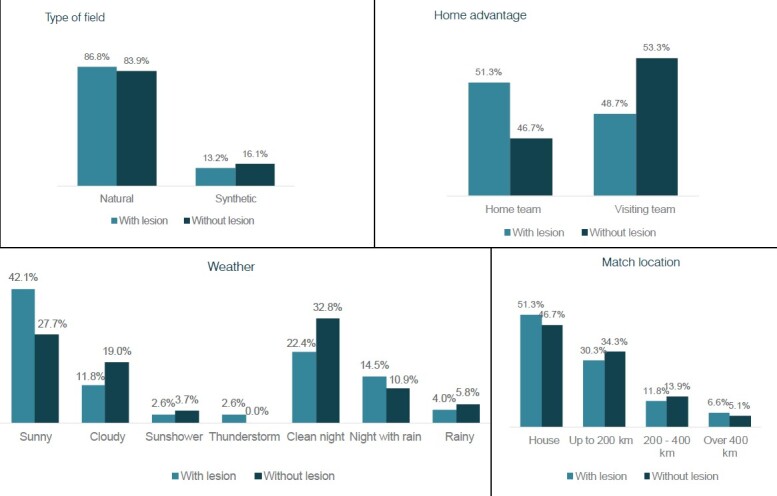
Comparison between the groups.

## CONCLUSION

The A1 series of the 2023 São Paulo Soccer Championship showed an incidence of 22.1 injuries per 1,000 hours of play. An increase of 33% when compared to the mean incidence in the pre-pandemic period (2016 to 2019).

## ANNEX

### Questionnaire:

Q1 – Which matches in the 2023 São Paulo Soccer Championship will you take part in?

Q2 – Which club are you answering for?

Q3 – What was the weather at the time of the match?

Q4 – What was the temperature at the time of the match?

Q5 – Were there any injuries during the match?

Q6 – Match location

Q7 – Who suffered the injury? (Date of birth)

Q8 – What is the athlete’s position?

Q9 – When did the injury occur? (Playing time)

Q10 – Did the injury occur after contact or collision with the ball or another player?

Q11 – If so, what was the circumstance?

Q12 – Was is considered a foul?

Q13 – What was the referee decision?

P14 – Injury site

P15 – Side of the lesion

P16 – Type of injury

P17 – Foot and Ankle

P18 – Leg

P19 – Knee

P20 – Pelvis, hip, and thigh

P21 – Trunk

P22 – Head

P23 – Shoulder

P24 – Other
